# Performance evaluation of rapid diagnostic tests for Chagas disease in Jutiapa, Guatemala

**DOI:** 10.1590/0037-8682-0065-2025

**Published:** 2025-09-29

**Authors:** Karla Lange, Andrea Marchiol, Rafael Herazo, Marianela Menes, Carlota Monroy, Antonieta Rodas, Belter Alcántara, Karen Carrillo, María Jesús Pinazo

**Affiliations:** 1 University of San Carlos of Guatemala, Faculty of Chemical Sciences and Pharmacy, School of Biological Chemistry, Guatemala City, Guatemala.; 2 Drugs for Neglected Diseases Initiative, Chagas Program, Rio de Janeiro, Brazil.; 3 University of San Carlos of Guatemala, Faculty of Chemical Sciences and Pharmacy, School of Biology, Guatemala City, Guatemala.

**Keywords:** Chagas Disease, Rapid diagnostic tests, Equity in access to health services.

## Abstract

**Background:**

Chagas disease remains a public health problem in Guatemala, with ongoing transmission, both regionally and globally. Diagnosis is complex and often inaccessible. This study evaluated the performance of rapid diagnostic tests for *Trypanosoma cruzi* infection in Jutiapa.

**Methods:**

Three rapid tests were performed, and the results were compared with those of a diagnostic algorithm for chronic infection*.*

**Results:**

Although all tests had high specificity, their sensitivities were suboptimal. The combined use of RDTs (Rapid Diagnostic Tests) slightly improved the sensitivity, but it remained below the ideal thresholds.

**Conclusions:**

Further evaluation of RDTs is necessary to enhance access and simplify diagnostic algorithms.

Guatemala is the Central American country with the highest burden of Chagas disease (CD), a significant public health problem. Although multiple efforts have been made to stop disease transmission, acute cases still persist, and positive cases are substantially underreported^1^.

In addition, access to diagnosis, treatment, and patient care for CD is limited by many factors^2^. Diagnosis is centralized at the National Health Laboratory, and in recent years, there has been slow progress in strengthening it through the decentralization and improvement of diagnostic tools. Progress has been made in the comprehensive clinical management and follow-up of positive cases. However, these capacities must be further improved and strengthened in endemic regions, and healthcare coverage has expanded to enable many individuals to be screened, diagnosed, and treated for CD. Timely diagnosis of *Trypanosoma cruzi* infection is crucial to enable treatment that can prevent or delay progression to the complications characteristic of the chronic phase of the disease.

Rapid diagnostic tests (RDTs), which detect anti-*T. cruzi* IgG antibodies, offer an alternative method for the serological diagnosis of chronic diseases. They provide rapid results and are easy to use, as they do not require complex equipment or specialized personnel^3^. According to a systematic review of the use of RDTs for CD, these tests demonstrate high sensitivity (Se) and specificity (Sp) during the chronic phase of the disease^4^. However, because their Se may be lower than that of ELISA (Enzyme-Linked Immunosorbent Assay), their effectiveness should be evaluated within each specific epidemiological setting in which they are implemented.

A few RDTs have been registered in Guatemala; however, their performance has not yet been validated. RDTs have the potential to bring diagnoses closer to communities and improve access to universal screening. 

Thus, it is necessary to evaluate the performance and operational characteristics of RDTs available for detecting anti-*T. cruzi* IgG antibodies in Guatemala. This would allow for the further development of a simplified diagnostic model for *T. cruzi* infection, which would help overcome access barriers.

The objective of this study was to evaluate, under field conditions, the performance of three RDTs registered in Guatemala in comparison with the currently used serological diagnostic algorithm. The aim was to improve access to the diagnosis of infections at the primary healthcare level. The study was conducted in the Department of Jutiapa, a high-risk area for disease transmission and the region with the highest prevalence in the country^2^.

A prospective observational study of diagnostic tests was conducted to assess the operational characteristics of three commercially available RDTs designed to detect anti-*T*. *cruzi* IgG antibodies. The performance of the RDTs was compared to that of ELISA, which forms part of the official serological algorithm in Guatemala^5^. The study was conducted from August to December 2022 in the Department of Jutiapa, Guatemala, in the villages of La Brea, within the municipality of Quesada, and within Ixcanal I and Ixcanal II in the municipality of Comapa. These rural villages have a mixed-race, Spanish-speaking population that is mainly engaged in agriculture. 

This study was conducted under field conditions during a community screening campaign. Samples for RDT processing were obtained via digital puncture, and the results were read on-site. Simultaneously, peripheral blood was collected for delayed serological processing.

Participants included individuals over one year of age who provided signed informed consent or assent (in the case of minors whose parents or guardians) and who had no history of trypanocidal treatment with nifurtimox or benznidazole.

The ONA^®^ program (Ona Systems Inc, designed to work with ODK collect, a free and open-source Android application, Burlington, VT 05401, USA) was used to store sociodemographic information of the individuals, and the ODK Collect application was used to store photographic records of the RDT cassettes used. Photographic recordings were obtained at the end of each reading session. An anonymized database was constructed using the collected data. 

Definitive serological diagnostic results were provided for each participant. Patients with confirmed *T. cruzi* infection were referred to the Jutiapa Health Area for evaluation, treatment, and follow-up, according to the Ministerio de Salud y Asistencia Social de Guatemala guidelines for patients with CD. The San Carlos University (USAC) Health Research Bioethics Committee approved this study. Bioethical endorsements were issued on April 21, 2022, with reference to adcobiusac 005-2022.

The method proposed by Li and Fine^6^ was used to calculate the necessary sample size, with N = 760, assuming a target Se of 0.97 and a prevalence of 10%. The analyzed operational characteristics were Se, Sp, accuracy, and kappa index (KI). 

For RDT processing, samples were obtained by capillary puncture, as indicated by each manufacturer. The RDTs were performed simultaneously. For conventional serological processing, samples were obtained via venous puncture. Serological assays for detecting anti*-T. cruzi* antibodies were performed at the Clinical Laboratory of the School of Chemistry and Pharmaceutical Sciences (USAC-Universidad de San Carlos, Guatemala, Brazil)*.* Quality control was performed at the Laboratorio Nacional de Salud (LNS).

Three RDTs with satisfactory performance, as reported in similar studies in other countries and registered and available in Guatemala, were selected^7^ ^,^ ^8^. The selection of RDTs included in the study considered their commercial availability in Guatemala (Table 1). Each RDT was read by two previously trained (blinded) operators according to the timings recommended by the manufacturer. In cases of disagreement, a third operator decided upon the case.

**TABLE 1:** Technical characteristics of the RDTs included in the study and reported by the manufacturers.

	Name	Manufacture/ Country	National Health registration	Viable (sample)	Volume WB (µL)	Reading time (min)	Required diluent (vol µL)	Se (%)*	Sp (%)*
On Site	Chagas AdBio Combo Rapid test cassette	CTK Biotech/ China	YES	S, P, WB	40 -50	15	70-100	92.9	100
Stat Pak	Chagas Stat Pak Assay	Chembio/USA	YES	S, P, WB	10	15	240	98.5	96
WL	WL Check Chagas	Wiener Lab/ Argentina	YES	S, P, WB	40	25-35	100	98.6	98.4

**RDT:** Rapid Diagnostic Tests, **S:** serum; **Se:** sensitivity; **Sp:** Specificity; **P:** plasma; **WB:** whole blood; *Reported by the manufacturer, **µ:** microliters.

For the official serological diagnostic algorithm, two ELISAs were used: initial lysate antigen (Chagatest ELISA lisado-Wiener-Argentina) and recombinant ELISA (Chagatest ELISA recombinant v.4.0, Argentina). Indirect hemagglutination (Chagatest HAI Wiener, Argentina) was performed in cases of discordance.

Statistical analysis of the sociodemographic characteristics of the screened individuals and the operational characteristics of the RDT was carried out using Epidat 3.1 software.

A parallel processing strategy was used to evaluate the performance of the RDTs. To estimate the diagnostic accuracy of the different combinations, three testing scenarios were explored: contingency tables were constructed, and accuracy, Se, and Sp were estimated with 95% confidence intervals (95% CI) using Epidat 3.1. In this step, false-positive rates were adjusted, and discrepancies were analyzed.

The performance of three RDTs was analyzed: OnSite (CTK Biotech, China), STAT PAK (Chembio, USA), and WL Check (Wiener, Argentina). These results were compared with the diagnostic reference standard established using the LNS algorithm. A total of 821 participants were included, of whom 547 were females (66.7%). Table 1 lists the performance parameters of each test.

The study showed that in individual analysis, the three tests evaluated had a limited Se between 71.76% and 77.91%, a high Sp between 95.76% and 99.43%, and substantial agreement with a KI between 0.687 and 0.806. (Table 2). The Se parameters observed in the three rapid tests were below 92% (95% CI), as recommended by PAHO ( Pan American Health Organization)^9^.

**TABLE 2:** Comparison of the performance of three rapid tests against the reference standard.

Test name	Se (%) 95% CI	Sp (%) 95% CI	Accuracy (%) 95% CI	LR+ 95% CI	LR- 95% CI	Odds ratio 95% CI	Kappa index
WL Check	71.8	99.4	96.5	125.9	0.3	446.7	0.8
Wiener	(63.4-83.6)	(98.8-100)	(95.2-97.8)	(48.6-348.6)	(0.2-0.4)	(150.1-1329.1)	(0.73-0.87)
STAT PAK	76.7	96.2	94.1	20.0	0.2	82.9	0.7
Chembio	(69.20-87.94)	(94.72-97.63)	(92.5-95.7)	(14.1-30.0)	(0.2-0.3)	(44.3-155.1)	(0.63-0.78)
OnSite CTK	77.9	95.8	93.8	18.4	0.2	79.6	0.68
Biotech	(67.84-86.92)	(94.04- 97.20)	(92.2-95.5)	(12.3-25.4)	(0.2-0.4	(42.5-149.0)	(0.6-0.76)

**Se:** Sensitivity; **Sp:** Specificity; **LR+:** positive likelihood; **LR:** negative likelihood; **CI:** confidence interval.

Using RDTs in parallel increased the performance and improved accuracy; however, the improvement in Se remained below 90% (Table 3).

**TABLE 3:** Performance of RDTs and their combinations in parallel.

Pairwise analysis	Se (%) CI 95%	Sp (%) CI 95%	Accuracy (%) CI 95%	Discordant (%)
OnSite CTK + STAT PAK	81.3 (70.7, 89.4)	98.5 (97.3-0.99)	96.8 (95.2-97.9)	5.8%
OnSite CTK + WL Check	80.0 (68.7, 88.6)	100 (99.4-100)	98.1 (96.8-98.9)	5.8%
STAT PAK + WL Check	80.0 (68.7, 88.6)	100 (99.5-100)	98,1 (96,9-99,0)	5.7%

**Se:** sensitivity, **Sp:** Specificity, **CI:** confidence interval.

This study provides evidence on the performance of three rapid tests for detecting chronic *T. cruzi* infection in communities within the Comapa Municipality, Jutiapa Health Area, which has the greatest burden of disease in Guatemala^4^. The rapid tests OnSite, STAT PAK, and WL Check were assessed under field conditions at the point of care by digital puncture for the RDT procedure and venous blood collection for the reference standard algorithm. 

In Guatemala, access to *T. cruzi* infection diagnosis remains a major barrier, primarily due to the limited availability of specialized equipment for serological processing (such as ELISA readers) in rural areas. This limitation significantly affects access to diagnosis and, consequently, equitable and timely treatment and comprehensive follow-up for patients. The use of RDTs in the country is still limited, and information on their performance is scarce. To the best of our knowledge, this study is the first to be systematized in Guatemala.

The Se results were similar for all three tests but fell below our expectations, with an average of 80%. However, the Sp were high (>95%).

The diagnostic accuracy of the RDTs in our study varied, ranging from 63.4 to 87.94% for Se and from 94.04 to 100% for Sp. This variability may be due to several factors in the parasite and human host. The parasite has significant genetic variability in the region, which, depending on the parasites in circulation, can result in immunoserological variability that affects the performance of RDTs^10^. The predominant DTU in this region was TcI, with infrequent circulation of TCII and TCIV^11^. However, the host immune response may vary due to different natural and/or pathological processes (immunosuppression, chronic disease, and etiological treatment, among others)^12^ ^,^ ^13^.

We can thus say that both STAT PAK (0.71) and OnSite CTK (0.71) exhibit good concordance (0.61-0.80), while WL Check, with a KI of 0.81, has better concordance; however, its CI falls below 0.8.

Our results differ from those obtained in other studies conducted in South American countries, where *T. cruzi* infection is endemic^14^ ^-^ ^16^. This discrepancy may be attributed to factors such as the epidemiology of the disease in the country and the strains (DTU) in circulation, resulting in variations in the current performance of these RDTs compared to that observed elsewhere^17^.

We modeled and analyzed the diagnostic performance of combinations of the three RDTs. As mentioned above, RDTs have good Sp (>95%) but limited Se (70-80%). RDT combinations were able to increase the Se to more than 80%, although this increase was not significant because a Se greater than 90%, which would be the recommended performance for systematic use, was not achieved. Modeling the use of two RDTs simultaneously (WL Check + STAT PAK) for 1,000 people with a 5% seroprevalence for CD allowed us to identify 42 people with *T. cruzi* infection. False positives are negligible due to the excellent Sp of these tests, which avoids erroneous indications for etiological treatment. Approximately 5% (51/1000) of the test results were discordant. This would lead to higher costs due to the need to refer the sample for conventional serology but would still allow for a conclusive result at a higher level in the health system. Finally, eight positive individuals were undiagnosed using these rapid tests (16%, 8/50 true positives) owing to their limited Se.

Other diagnostic kits are available on the market, but they are not yet available in Guatemala. Therefore, it is necessary to continue validating other RDTs, including those from the international market, which have shown excellent performance in other areas. This study was conducted in Jutiapa, one of the most endemic areas in Guatemala. However, examining other areas of the country to verify whether the performance of RDTs is similar is important. The use of rapid tests is crucial for advancing the diagnosis, treatment, and elimination of CD as a public health issue, in accordance with the new recommendations published by PAHO^18^.

A cost-effectiveness study on the use of RDTs in combination is needed to contribute to the creation of evidence and an impact evaluation that will provide recommendations for the country.

The current and future use of rapid tests is important for health services, particularly in primary healthcare settings, as they facilitate and enhance the implementation and efficiency of healthcare. Despite these limitations, these tests are simple and accessible, enabling the identification of populations infected with *T. cruzi* at the community level. As the number of diagnosed individuals increases through the use of RDTs, health services must intensify their efforts to ensure treatment and subsequent follow-up. Without this commitment, adequate access to care for affected populations will remain unattainable.

## Data Availability

After publication the data will be available on demand to authors.
